# Effect of Embryo Vitrification on Rabbit Foetal Placenta Proteome during Pregnancy

**DOI:** 10.1371/journal.pone.0125157

**Published:** 2015-04-27

**Authors:** Maria Desemparats Saenz-de-Juano, José Salvador Vicente, Kristin Hollung, Francisco Marco-Jiménez

**Affiliations:** 1 Instituto de Ciencia y Tecnología Animal, Universidad Politècnica de València, Valencia, Spain; 2 Nofima AS, Aas, Norway; Medical Faculty, Otto-von-Guericke University Magdeburg, Medical Faculty, GERMANY

## Abstract

Very limited information on the post-implantatory effects of vitrification has been published till now. We observed in a previous study that the vitrification procedure for the cryopreservation of embryos introduced transcriptomic and proteomic modifications in the rabbit foetal placenta at the middle of gestation. Now, we have conducted a proteomic study to determine whether protein alterations in the foetal placenta induced by the vitrification procedure remain during pregnancy. In this study, we used 2D-DIGE and mass spectrometry (MALDI-TOF-TOF and LC-MS/MS analysis) to identify the protein changes during middle and late stages of gestation (Day 14 and Day 24, respectively) in rabbit foetal placenta. We identified 11 differentially expressed proteins at Day 14 and 13 proteins at Day 24. Data are available via ProteomeXchange with identifiers PXD001840 and PXD001836. In addition, we demonstrate the presence of three proteins, serum albumin, isocitrate dehydrogenase 1 [NADP+], and phosphoglycerate mutase 1, which were altered during pregnancy. We demonstrate the existence of changes in foetal placental protein during pregnancy induced by the vitrification procedure, which brings into question whether vitrification effects observed during foetal development could lead to physiological and metabolic disorders in adulthood. This effect, taken together with other effects reported in the literature, suggests that embryo cryopreservation is not neutral.

## Introduction

Vitrification was introduced in 1985 as a simple and cheap method to cryopreserve mammalian embryos in the absence of ice [1]. Since then, vitrification is replacing slow freezing procedure as the most popular method for embryo storage [2].

It is known that vitrification could be harmful to embryos, but is not considered to affect survivors, which are regarded as neutral [3]. For this reason, up to now most of the works that aimed to understand the effects of vitrification were performed in pre-implantatory embryos. In consequence, very little information is available about effects on post-implantatory development. In rabbits, it has been observed that there is an important peak of losses after Day 14 of development [4–7]. That means that vitrification damage is not completely removed after implantation and not all implanted embryos are able to reach the end of gestation. In a previous study, we demonstrated that vitrification induced a reduction in foetal and placental development between Day a 10 and 14 of gestation [6], and later we related those alterations with modifications in gene and protein expression [7]. It has been proposed that the alterations caused by a hazardous developmental environment are more easily restored in tissues derived from the inner cell mass than in those resulting from the trophectoderm such as the placenta. In addition, it was also suggested that variations in the intrauterine availability of nutrients, oxygen and hormones could give rise to abnormalities and diseases that may persist into adulthood [8].

In our previous study, we reported that vitrification procedure for the cryopreservation of embryos introduced transcriptomic and proteomic modifications in rabbit foetal placenta at the middle of gestation (Day 14). However, there is no report to determine if proteomic changes induced by the vitrification procedure in foetal placental still remained during pregnancy. In the present investigation, we report the proteome dynamics of rabbit placenta isolated from vitrified embryos during different stages of gestation, at the middle (Day 14) and end (Day 24). This study shows, for the first time, that the proteome alterations remained during gestation. The study provides the first description of proteome alterations during gestation induced by vitrification procedure. This raises the question of whether vitrification effects observed during foetal development could lead to physiological and metabolic disorders in adulthood.

## Materials and Methods

### Animals

A total of 22 New Zealand White rabbit does from the ICTA (Instituto de Ciencia y Tecnología Animal) at the Universidad Politécnica de Valencia (UPV) were used as donors and recipients. All animals were handled according to the principles of animal care published by Spanish Royal Decree 53/2013 and approved by the UPV Research Ethics Committee.

### Embryo collection

Twelve donor does were artificially inseminated with pooled sperm from fertile males and euthanised at 72 hours post-insemination with an intravenous injection of 200 mg/Kg of pentobarbital sodium (Dolethal, Vétoquinol, Madrid, España). Embryos were recovered by perfusion of each oviduct and uterine horn with 10 mL pre-warmed Dulbecco Phosphate Buffered Saline (DPBS; Sigma-Aldrich, Madrid, Spain) supplemented with 0.2% of Bovine Serum Albumin (BSA; Sigma-Aldrich, Madrid, Spain). Morphologically normal embryos were distributed into pools of 15 embryos for fresh transfer or vitrification.

### Vitrification and warming procedure

Embryos were vitrified using the methodology described by Marco-Jiménez et al. (2013), which contains two steps at 20°C. In the first step, embryos were placed for 2 min in a vitrification solution consisting of 12.5% dimethyl sulphoxide (DMSO; Sigma-Aldrich, Madrid, Spain) and 12.5% ethylene glycol (EG; Sigma-Aldrich, Madrid, Spain) in DPBS supplemented with 0.2% of BSA. In the second, embryos were suspended for 30 seconds in a solution of 20% DMSO and 20% EG in DPBS supplemented with 0.2% of BSA. After warming in a water bath at 20°C for 10–15 seconds, the vitrification medium was removed while loading the embryos into a solution containing DPBS and 0.33 M sucrose (Sigma-Aldrich, Madrid, Spain) for 5 min, followed by one bath in a solution of DPBS for another 5 min. Only non-damaged embryos, with homogenous blastomeres, intact mucin coat and zona pellucida were included in the study.

### Embryo transfer by laparoscopy

A total of 68 fresh and 75 vitrified morphologically normal embryos were transferred into oviducts by laparoscopy to 10 recipient does (13 to 15 embryos per recipient) following the procedure described by Besenfelder and Brem [9]. Ovulation was induced in recipient does with an intramuscular dose of 1 mg of Buserelin Acetate (Suprefact, Hoechst Marion Roussel S.A, Madrid, Spain) 68–72 hours before transfer.

To sedate the does during laparoscopy, anaesthesia was administered by an intramuscular injection of 5 mg/Kg of xylazine (Bayer AG, Leverkusen, Germany), followed 5–10 min later by an intravenous injection into the marginal ear vein of 6 mg/Kg of ketamine hydrochloride (Imalgène, Merial SA, Lyon, France). During laparoscopy, 3 mg/kg of morphine hydrochloride (Morfina, B. Braun, Barcelona, Spain) was administered intramuscularly. After transfer, does were treated with antibiotics (4mg/Kg of gentamicin every 24h for 3 days, 10% Ganadexil, Invesa, Barcelona, Spain) and analgesics (0.03mg/Kg of buprenorphine hydrochloride, [Buprex, Esteve, Barcelona, Spain] every 12 hours for 3 days and 0.2mg/Kg of meloxicam [Metacam 5mg/mL, Norvet, Barcelona, Spain] every 24h for 3 days).

In order to remove the maternal effect, both types of embryos were transferred to the same female (6–8 embryos of each type per oviduct).

### Tissue recovery, protein extraction, and quantification

**Fig 1:**
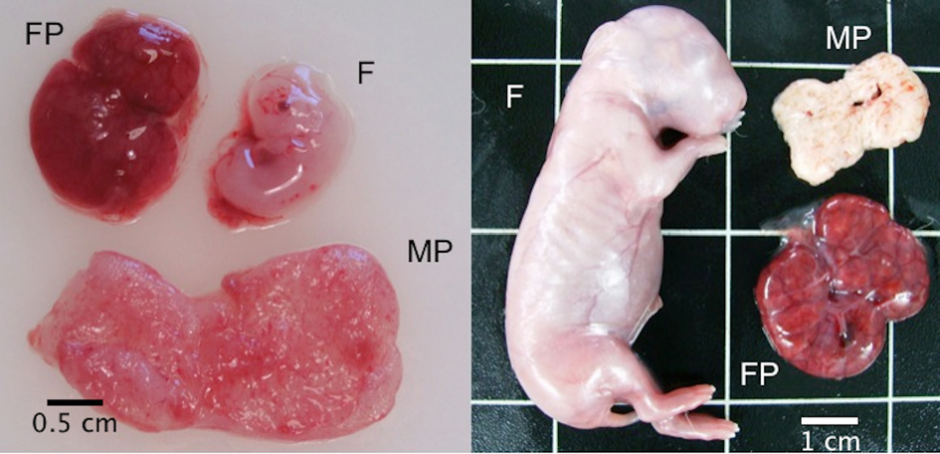
Rabbit foetus (F), foetal placenta (FP) and maternal placenta (MP) at Day 14 (left) and Day 24 (right) of gestation.

Eleven days after the transfer (Day 14 of gestation), 6 recipient does were euthanised with an intravenous injection of 200 mg/Kg of pentobarbital sodium (Dolethal, Vétoquinol, Madrid, España) and a portion of 14-day-old foetal placenta was recovered (Fig 1A). A total of 16 samples (8 for each experimental group) were collected for proteomic analysis. The same procedure was performed twenty-one days after transfer (Day 24 of gestation) in the remaining 4 recipients (Fig 1B). Data on foetus, foetal and maternal placentas weights were collected.

Proteins were extracted from foetal placental tissue by sonication in 500 μL of RIPA buffer (150 mM NaCl, 1.0% IGEPAL CA-630, 0.5% sodium deoxycholate, 0.1% SDS, and 50 mM Tris, pH 8.0; Sigma-Aldrich, Madrid, Spain) combined with an anti-protease enzyme. Then, samples were incubated on ice for 20 min and centrifuged at maximum speed for a further 20 min. Supernatant were collected and total protein was quantified using the BCA Protein Assay Kit (Thermo Scientific, Madrid, Spain) using BSA as standard.

### Fluorescent protein labelling and 2D DIGE analysis

The Two-Dimensional Gel Electrophoresis using DIGE labelling of proteins was carried out as previously described [10, 11]. A volume of protein sample equivalent to 50 μg was labelled with 400 pmol of CyDye (GE Healthcare Bio-Sciences AB, Uppsala, Sweden). Each sample was labelled both with Cy3 and Cy5 as technical replicates and run on separate gels. A reference was made as a mix of all samples, labelled with Cy2 and separated on all gels. The sample was vortexed and left on ice for 30 min in the dark. The reaction was stopped by adding 1μl 10 mM lysine, then the sample was vortexed and left on ice for 10 min in the dark. An equal volume of TES buffer was added after the protein sample was labelled. The sample was vortexed and left on ice for 10 min. Then, differentially labelled samples were mixed. All samples were randomised prior to separation. IPG strips (24 cm) spanning the pH region 5–8 were used in the first dimension. For analytical 2-DE, 150 μg pre-labelled proteins were loaded onto each IPG strip by in-gel rehydration, while 500 μg proteins were loaded for preparative 2-DE. The isoelectric focusing was performed on the Pharmacia Multiphor unit equipped with a temperature controller (GE Healthcare). Low voltage (50 V) was applied in the initial step, followed by a stepwise increase to 3500 V and reaching a total of 70 000 Vh. Proteins were separated on 12.5% SDS-PAGE in the second dimension using the Ettan Dalt *twelve* Large Format Vertical System (GE Healthcare). After SDS-PAGE, CyDye- labelled gels were scanned directly using the Ettan DIGE Imager (GE Healthcare). The excitation wavelengths for Cy2, Cy3 and Cy5 were 480 nm, 540 nm and 635 nm, and the emission wavelengths were 530 nm, 595 nm and 680 nm, respectively. The preparative gels were silver stained according to Shevchenko et al. [12]. Six preparative gels were made for repeated identification of the proteins for each type of sample (14 and 24-day-old foetal placentas). The images from the scanned gels were imported to Progenesis SameSpots v4.5 (Nonlinear Dynamics). A reference gel was selected to match all other gels, and artefacts and mismatched spots were removed by manual editing. The protein spots were matched throughout all 16 gels, and the spot volumes, defined as the ratio between the amount of protein measured in the reference image and the amount of the same protein measured in the sample image, were normalised by dividing the raw volume of each spot in a gel by the total volume of valid spots in that gel. Normalised spot volumes were used to compare different groups statistically and to determine fold-change values. An ANOVA was carried out in the Progenesis SameSpots v4.5 software and a False Discovery Rate (FDR) analysis was used to calculate adjusted p-values (q-value). Finally, in order to improve reliability and consistency of the analysis, the normalised volumes resulting table was imported into Unscrambler version 10.2 (CAMO A/S, Norway) and a Principal Component Analysis (PCA) and a Partial Least Square (PLS) regression analysis were performed. The significance level for all of the analyses was set as 0.05.

### Protein identification

#### MALDI-TOF/TOF

The protein spots of interest were punched out of the preparative gels using pipette tips and extracted from gels according to the method of Jensen et al. [13]. Briefly, the washing/dehydration process was carried out by adding 150 μl of 50% v/v acetonitrile (ACN) and shaking for 15 min at room temperature before the gels were dried in a speed-vac centrifuge (ISS 110 SpeedVac System, Thermo Savant) for 30 min. Then, 150 μl of 10 mM DTT was added and the gel plugs were incubated for 45 min at 56°C, followed by addition of 150 μl 55 mM iodoacetamide and incubated for 30 min at room temperature in the dark. Afterwards, the plugs were washed with 50% v/v ACN and dried. 30 μl trypsin digestion buffer (5 ng/ μl) was added to the dried gel pieces, incubated on ice for 30 min, and at 37°C overnight. A C18 column was packed in a GeLoader tip (Eppendorf, Hamburg, Germany). A 10 ml syringe was used to force liquid through the column. 20 μl of the tryptic protein digests were loaded onto the column, and washed with 20 μl of 0.1% Trifluoroacetic acid (TFA). The peptides were eluted with 0.8 μl matrix solution (5 mg/ml alpha-cyano-4-hydroxy-trans-cinnamic acid (Agilent Technologies, Inc.) in 70% ACN/0.1% TFA) and spotted directly onto the MALDI plate. The identification of the samples spotted on the MALDI plate was performed with an Ultraflex MALDI-TOF/TOF mass spectrometer with the LIFT module (Bruker Daltonics). The software employed for the data analysis was FlexAnalysis 2.4 package (Version 1.1.3, Bruker Daltonics), BioTools 3.0 (Version 1.0, Bruker Daltonics) and MASCOT (http://www.matrixscience.com/) database search program. An accuracy of 100 ppm (parts per million) was used in the search criteria. Fixed modifications and variable modification used were carbamidomethyl (C) and oxidation (M), respectively. MS/MS analysis and repeated Mascot-based database searches of minimum three precursor ions recognised in the peptide mass fingerprint (PMF) search were performed to confirm the PMF-based protein identification. The mass spectrometry proteomics data have been deposited in the ProteomeXchange Consortium [14] via the PRIDE partner repository with the dataset identifier PXD001840.

#### LC-MS/MS

Several spots that were not identified with the MALDI-TOF/TOF were re-analysed with LC-MS/MS. A volume of 5 μl of sample were loaded onto a trap column (NanoLC Column, 3 μ C18-CL, 350 um x 0.5 mm; Eksigen) and desalted with 0.1% TFA at 3 μl/min during 5 min. The peptides were loaded onto an analytical column (LC Column, 3 μ C18-CL, 75 um x 12 cm, Nikkyo) equilibrated in 5% ACN, 0.1% formic acid (FA). Elution was carried out with a linear gradient of 5 to 45% B in A for 15 min (A: 0.1% FA; B: ACN, 0.1% FA) at a flow rate of 300 ηl/min. Peptides were analysed in a mass spectrometer nanoESI qQTOF (5600 TripeTOF, ABSCIEX). ProteinPilot default parameters were used to generate peak list directly from 5600 TripleTOF wiff files. The Paragon algorithm of ProteinPilot was used to search the Expasy protein database with the following parameters: trypsin specificity, iodoacetamide cys-alkylation, taxonomy restricted to *Homo sapiens*, and the search effort set to rapid. To avoid using the same spectral evidence in more than one protein, the identified proteins were grouped based on MS/MS spectra by the ProteinPilot Progroup algorithm. The mass spectrometry proteomics data have been deposited in the ProteomeXchange Consortium [14] via the PRIDE partner repository with the dataset identifier PXD001836.

### Functional analysis

The rabbit sequences of the identified proteins were uploaded to BLAST2GO software version 2.8 [15], and functional annotation was performed using BLASTP against NCBI nr-with default parameters. The list of the identified protein was also subjected to STRING software version 9.1 [16] to reveal functional interactions between the altered proteins. Enrichment analysis for specific Gene Ontology (GO) categories for biological process, molecular function and cellular components was performed using default parameters and the *Homo sapiens* database.

### Statistical analysis

Data relative to foetus, foetal placenta and maternal placenta weight were analysed using a General Linear Model (GLM). Analyses were performed with SPSS 16.0 software package (SPSS Inc., Chicago, Illinois, USA, 2002). Values were considered statistically different at P<0.05. Results are reported as least square means with standard error of the mean (SEM).

## Results

### Effect of vitrification of foetal and placental weights

As Table 1 shows, at Day 14 there were significant differences in foetus and maternal placenta weights between control and vitrified embryos (P<0.05). However, when we compared the foetal and placental weights at Day 24 we did not detect any significant difference between experimental groups (P>0.05).

**Table 1:** Foetal and placental weights at Day 14 and Day 24 of gestation.

Day	n	Embryo Type	Foetus (g)	Foetal Placenta (g)	Maternal Placenta (g)
**14**	25	Control	0.30 ± 0.016	0,57 ± 0.033	1.18 ± 0.059
	29	Vitrified	0.24 ± 0.016*	0.52 ± 0.030	0.89 ±0.054 *
**24**	22	Control	13.42 ± 0.550	3.13 ± 0.160	1.87 ± 0.113
	19	Vitrified	12.70 ± 0.592	3.26 ± 0.172	1.73 ± 0.122

n = number of samples.

* indicates significant differences between control and vitrified embryos (P<0.05).

### Effect of vitrification on foetal placenta proteome at two different times of gestation

In the present study, the proteome of control and vitrified foetal placenta was compared at two different times of post-implantatory development: Day 14 and Day 24. To determine the differential proteins, a 2D-DIGE analysis followed by MALDI-TOF/TOF or LC-MS/MS was performed. 2D-DIGE analyses of foetal placenta protein extracts were performed using eight biological replicates for each experimental group. In addition, a technical replicate was also carried out for each sample, changing the dye type of the sample. In order to be more specific, a narrow isoelectric point range (pH 5–8) was selected. A total of 1043 spots were matched across all the gels in 14-day-old foetal placentas and 1275 spots in 24-day-old foetal placentas.

**Fig 2:**
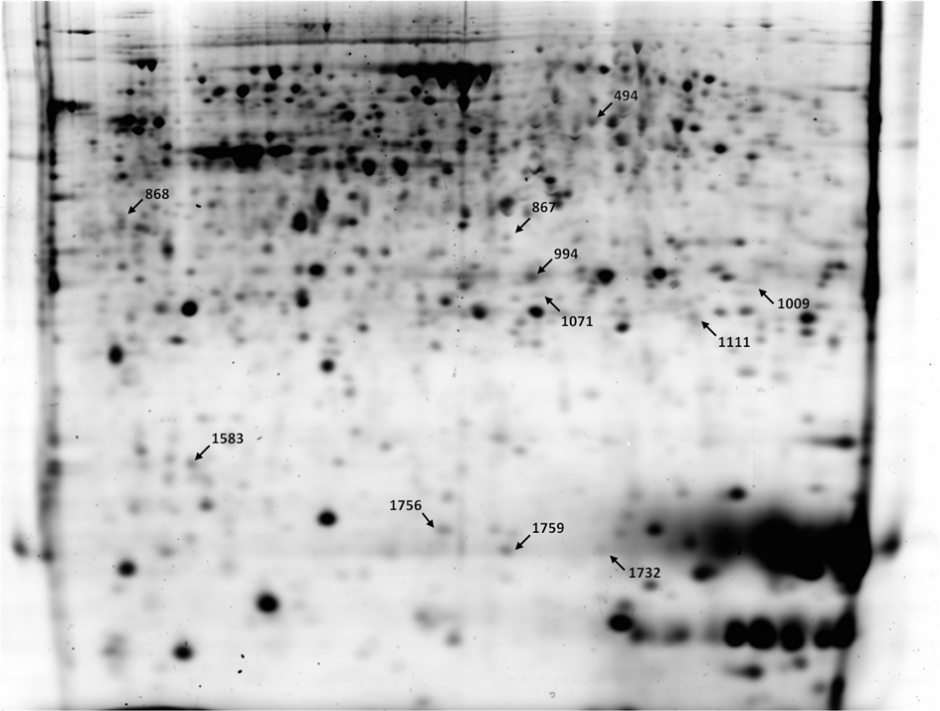
Representation of 2D-DIGE gel at Day 14. Proteins picked for identification are outlined with an arrow and the tagged numbers correspond to the same ones indicated in Table 2.

In the case of 14-day-old foetal placenta, 100 spots were considered statistically significant after ANOVA analysis. From that list, 16 protein spots (8 upregulated and 8 downregulated) were also detected as significant by PLS regression analysis. We considered those 16 altered protein spots as the most interesting for further identification. Finally, 11 spots were successfully identified and are shown in Fig 2. The list of identified proteins and detailed information of them are provided in Table 2. In 14-day-old foetal placenta from vitrified embryos, higher amounts were observed of serum albumin precursor (ALB), phosphoglycerate mutase 1 (PGAM1), haemoglobin subunit alpha (HBA), haemoglobin subunit beta (HBB) and transthyretin (TTR). Lower amounts were observed of proliferation-associated protein 2GA (PA2G4), ATP synthase (ATP5A1), heat shock protein 90kDa alpha class A member 1 (HSP90AA1), heat shock protein 90kDa beta member 1 (HSP90B1), isocitrate dehydrogenase 1 [NADP+] (IDH1), and chaperonin containing TCP1 subunit 2 beta (CCT2).

**Table 2:** List of identified altered proteins at Day 14 of gestation.

Spot n°.	Gene name	Accession n° Ensembl or NCBI	Fold Change	Mol. Mass (Theoretical)	pI (Theoretical)	Peptide matched	Seq. Cov. (%)	Description
494	PA2G4	ENSOCUP00000014901	-1.15	43543	6.2	15	40	Proliferation-associated protein 2G4
867	ATP5A1	ENSOCUP00000003441	-1.29	59753	9.5	10	34	ATP synthase, H+ transporting, mitochondrial F1 complex, alpha subunit 1, cardiac muscle
868	HSP90AA1	ENSOCUP00000001106	-1.19	78339	4.6	9	12	Heat shock protein HSP 90-alpha
994	ALB	ENSOCUP00000014006	1.64	68965	6.3	16	30	Albumin
1009	PGAM1	ENSOCUP00000005679	1.42	23649	7.0	23	52	Phosphoglycerate mutase 1
1071	HSP90B1	ENSOCUP00000014008	-1.47	89314	4.6	5	9	Heat shock protein 90kDa beta (Grp94), member 1
1111	IDH1	ENSOCUP00000002310	-1.19	46895	7.0	5	14	Isocitrate dehydrogenase 1 (NADP+), soluble
1583	CCT2	ENSOCUP00000014783	-1.34	57458	6.4	7	16	Chaperonin containing TCP1, subunit 2 (beta)
1732	HBA1	P1948	1.41	15589	8.3	1	11	Haemoglobin subunit alpha
1756	HBB	ENSOCUP00000000491	1.45	16141	8.2	3	18	Haemoglobin subunit beta
1759	TTR	ENSOCUP00000014907	1.32	15754	5.9	2	20	Transthyretin

Mol. Mass: Molecular Mass; pI: Isoelectric point; Seq. Cov.: sequence coverage.

**Fig 3:**
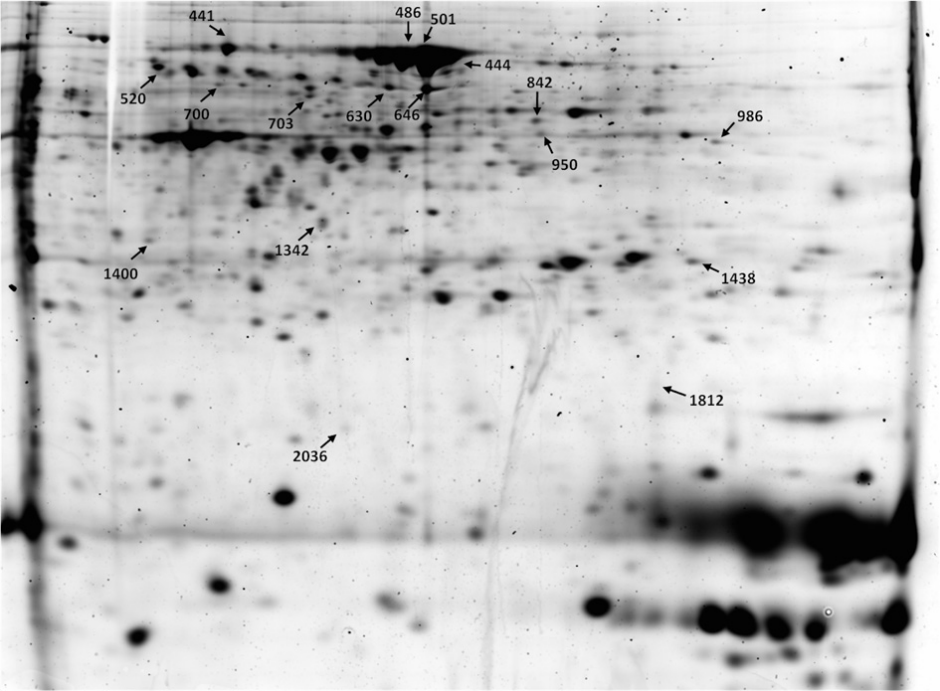
Representation of 2D-DIGE gel at Day 24. Proteins picked for identification are outlined with an arrow and the tagged numbers correspond to the same ones indicated in Table 3.

In the case of the 24-day-old foetal placenta, ANOVA analysis detected 254 differentially expressed protein spots between vitrified and control groups. From that list, 32 protein spots (23 upregulated and 9 downregulated) were also detected as significant by PLS regression analysis. We considered those 32 altered protein spots as the most interesting for further identification. From those protein spots, 4 were removed from the final identification selection due to their huge molecular weight and a total of 28 protein spots were picked from preparative gels for protein identification. Finally, only 8 were identified using MALDI-TOF/TOF analysis and 11 spots by LC-MS/MS. We successfully identified 17 protein spots, but some of the spots were identified as the same protein, such as in the case of albumin (ALB), protein disulfide-isomerase A3 (PDIA3) and heat shock protein 8 (HSPA8). In total, 13 unique proteins were identified. Successfully identified proteins are indicated in Fig 3 and Table 3. In placentas derived from vitrified embryos, higher amounts were observed of alpha-1-antiproteinase F (SERPINA1), protein disulfide-isomerase A3 (PDIA3), adenosine kinase isoform X4 (ADK), phosphoglycerate mutase 1 (PGAM1), heat shock 70 kDa protein 5 (HSPA5), isocitrate dehydrogenase 1 [NADP+] (IDH1), heat shock protein 8 (HSPA8), Pyrophosphatase (PPA1), prolyl 4-hydroxylase beta polypeptide (P4HB) and stathmin 1 (STMN1). Lower amounts were observed of serum albumin precursor (ALB) and vacuolar protein sorting 29 (VPS29).

**Table 3:** List of identified altered proteins at Day 24 of gestation.

Spot n°.	Gene name	Accession n° Ensembl or NCBI	Fold Change	Mol. Mass (Theoretical)	pI (Theoretical)	Peptide matched	Seq. Cov. (%)	Description
441	HSPA8	ENSOCUP00000013344	1.85	71082	5.1	18	26	heat shock protein 70 cognate
444	ALB	ENSOCUP00000014006	-1.85	68965	6.3	12	22	serum albumin precursor
486	ALB	ENSOCUP00000014006	-1.95	68965	6.3	18	38	serum albumin precursor
501	ALB	ENSOCUP00000014006	-1.85	68965	6.3	13	28	serum albumin precursor
520	SERPINA1	ENSOCUP00000015803	1.91	45796	6.1	15	39	alpha-1-antiproteinase F
630	PDIA3	ENSOCUP00000001003	1.94	56572	6.0	40	66	protein disulfide-isomerase A3
646	PDIA3	ENSOCUP00000001003	1.95	56572	6.0	23	45	protein disulfide-isomerase A3
700	ACTB	ENSOCUP00000013344	1.22	36272	5.6	19	42	actin, cytoplasmatic 1
703	HSPA5	ENSOCUP00000025421	1.38	72435	4.9	52	44	heat shock 70 kDa protein 5
842	ALB	ENSOCUP00000014006	1.63	68965	6.3	49	47	serum albumin precursor
950	ADK	ENSOCUP00000008569	1.74	40404	6.8	15	48	adenosine kinase
986	IDH1	ENSOCUT00000002310	1.45	46895	7.0	24	47	Isocitrate dehydrogenase [NADP+] cytoplasmic
1342	PPA1	ENSOCUP00000013751	2.02	30810	5.7	12	31	pyrophosphatase 1
1400	P4HB	P21195	1.42	56808		11	14	Prolyl 4-Hydroxylase, Beta Polypeptide
1438	PGAM1	ENSOCUP00000005676	4.48	23649	7.0	19	70	phosphoglycerate mutase 1
1812	VPS29	ENSOCUP00000026682	-1.49	20917	7.0	2	13	vacuolar protein sorting 29
2036	STMN1	ENSOCUP00000005611	1.59	17171	5.8	14	59	Stathmin

Mol. Mass: Molecular Mass; pI: Isoelectric point; Seq. Cov.: sequence coverage.

Comparing the altered proteins at Day 14 and Day 24, we found that there were three proteins that remained altered at both times: ALB, IDH1 and PGAM1. ALB changed from upregulated at Day 14 to downregulated at Day 24. The opposite happened to IDH1, which changed from downregulated to upregulated. PGAM1 remained upregulated in both times, being the most overexpressed protein at Day 24.

### Functional distribution of identified altered proteins

To interpret the data more easily and efficiently, we used a GO analysis to evaluate the localisations and functions of the altered proteins. Functional annotation and enrichment analysis for specific GO categories for biological process, molecular function and cellular component were achieved using BLAST2GO and STRING software.

**Fig 4:**
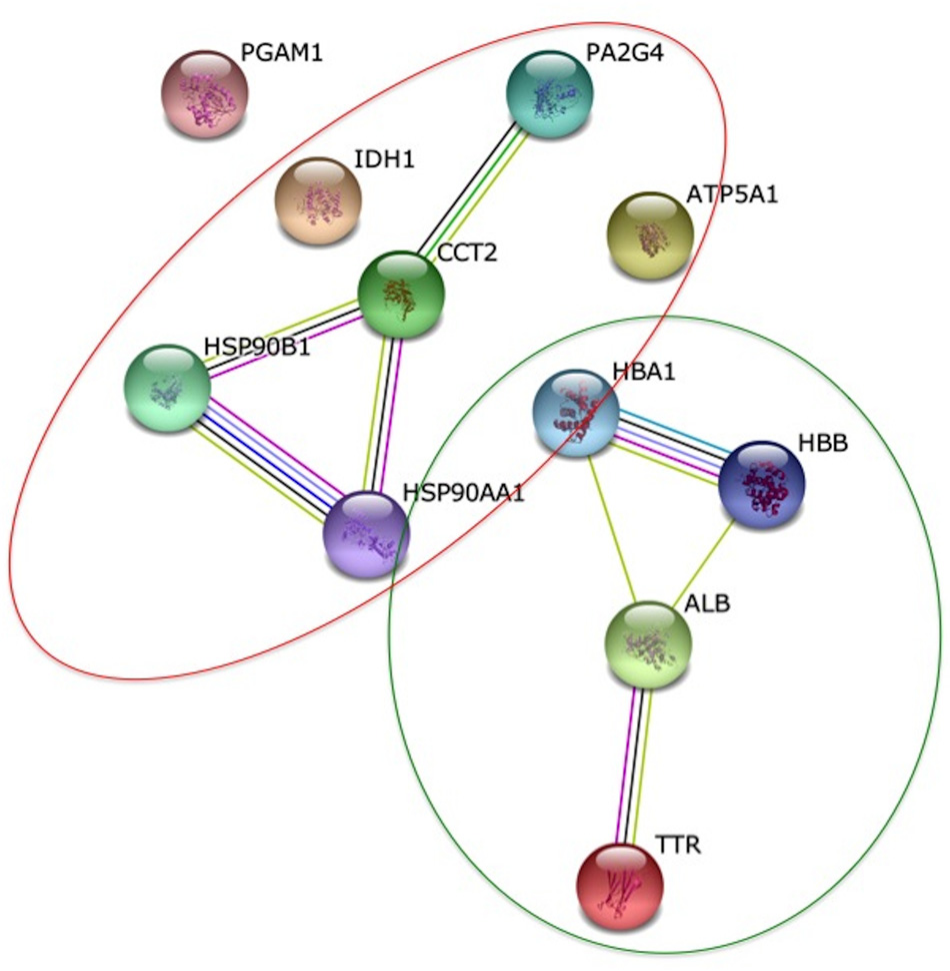
Protein interaction network of altered proteins at Day 14. In the network generated by STRING v.9.1, each node represents a protein and each edge represents an interaction, coloured by evidence type (see STRING website for colour legend). The original graphic output was modified including circles to group the proteins according to their regulation. A green line means upregulation and a red line downregulation.

In the case of 14-day-old foetal placenta, we found that the six downregulated proteins were mainly involved in primary metabolic processes, cellular metabolic processes and organic substance metabolic processes. As regards molecular function, they were mainly related with ion and nucleotide binding. Significantly biological processes overrepresented in the five upregulated proteins were oxygen transport, gas transport and hydrogen peroxide catabolic process. Molecular functions significantly overrepresented were oxygen binding, haptoglobin binding, antioxidant activity, peroxidase activity and oxidoreductase activity. When we evaluated the networks between altered proteins, we found that there were two separate relations: one between upregulated proteins and another between the downregulated proteins (Fig 4).

**Fig 5:**
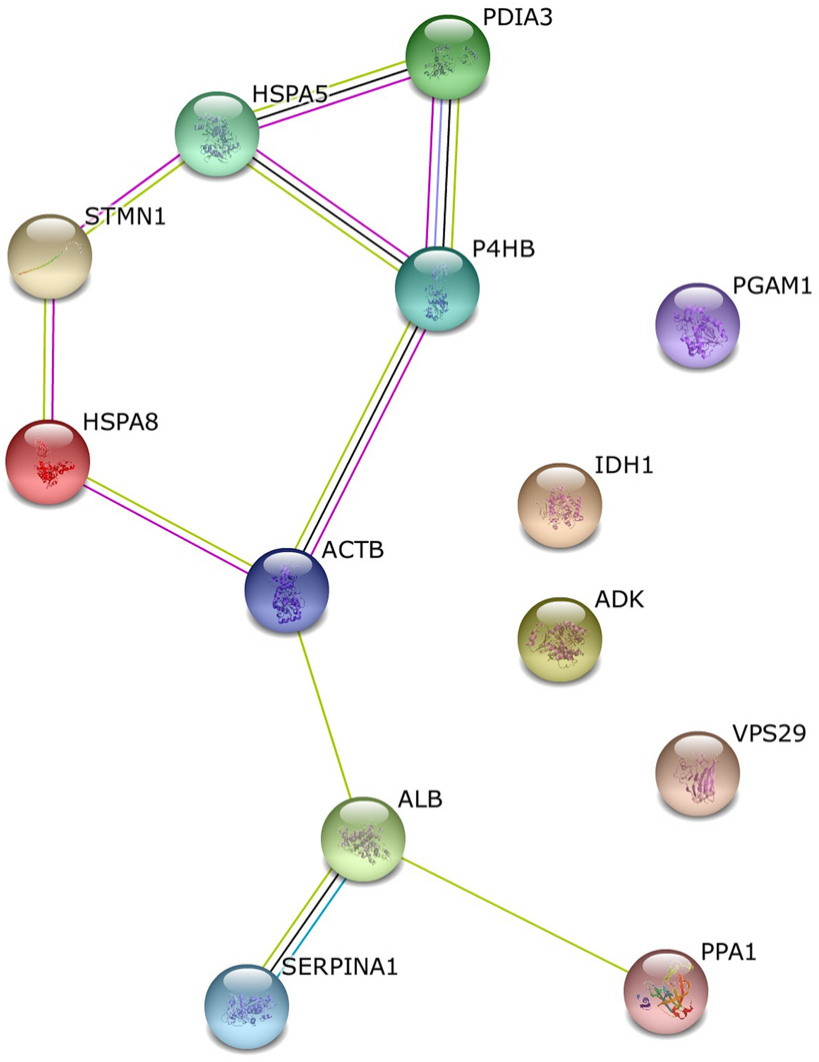
Protein interaction network of altered proteins at Day 24. In the network generated by STRING v.9.1, each node represents a protein and each edge represents an interaction, coloured by evidence type (see STRING website for colour legend).

In the case of 24-day-old foetal placenta, we found that there were two KEGG pathways significantly altered: antigen processing and presentation and protein processing in the endoplasmatic reticulum. The most general biological processes affected were regulation of cellular processes and small molecule metabolic processes. As far as molecular function is concerned, the altered categories were binding (nucleotide, metal ion, anion and protein), oxidoreductase and isomerase activity. Moreover, the most represented and significantly overrepresented cellular component term was extracellular vesicle exome, with 9 proteins from the 12 identified. The network generated with STRING that connected altered proteins is shown in Fig 5.

## Discussion

Up to now, very few studies have investigated the effects of vitrification of embryos on placenta development [4, 6, 7]. In a previous work, we associated vitrification procedure with low foetal size and weight at mid-gestation (Day 14) [7]. Additionally, transcriptomic and proteomic modifications in foetal placenta were also found. Hence, in the current study we conducted a proteomic analysis to determine whether vitrification of embryos induces proteomic modifications on the foetal placenta that remain during pregnancy.

Since placenta is the critical channel between the mother and foetus for the transportation of oxygen and nutrients, it can be explained why the largest proportion of proteins identified at Day 14 were linked to metabolic pathway and gas transport. In particular, proliferation-associated protein 2G4 (PA2G4) is implicated in cell growth, apoptosis and differentiation, and it has been observed that deletion of this protein leads to foetal growth retardation [17]. Haemoglobin alpha (HBA1) and beta (HBB) was upregulated in placenta of women exposed to cigarette smoking during pregnancy followed by a higher risk of preterm delivery [18]. An upregulation of transthyretin protein (TTR) was also observed in our previous transcriptomic analysis [7]. TTR appears to play an important role in the delivery of maternal thyroid hormone to the developing foetus. Interestingly, the mRNA expression of this protein was also upregulated in pre-eclamptic placentas [19] and in cases of intrauterine growth restriction [20]. Finally, comparing the identified proteins with those previously identified at Day 14 [7], protein serum albumin (ALB) and HBB were coincident.

Due to the biological function of altered transcripts and proteins that we observed in foetal placentas at Day 14, it could be supposed that all these changes in cellular physiology might be responsible of the lower weight of foetus and maternal placenta observed, and might implicate the post-implantational losses observed between Day 14 and birth. As Mocé et al. [4] observed, most of the foetal mortality due to embryo vitrification occurs between day 8 and 17 of gestation, meaning that foetal losses usually take place soon after implantation. That could explain why in the current work no differences in foetal or placental weights were found at Day 24, suggesting that those embryos with altered growth failed before this point.

As we aimed to determine whether the proteomic differences previously observed at Day 14 were temporary or not, we decided to analyse the proteome of foetal placentas at Day 24. Then, we detected 32 significantly altered proteins and from this list 17 were successfully identified. The altered proteins were mainly related with antigen processing, presentation and protein processing in endoplasmatic reticulum, regulation of cellular process and small molecule metabolic process.

From the list it is important to highlight the presence of alpha-1-antiproteinase F (SERPINA1) related with Complement and Coagulation Cascades, highly altered in the transcriptomic analysis at Day 14 [7]. In addition, ALB, phosphoglycerate mutase 1 (PGAM1) and isocitrate dehydrogenase 1 [NADP+] (IDH1) were also previously detected as altered in vitrified embryos at Day 14. While ALB and IDH1 was expressed in the opposite direction, PGAM1 remained upregulated, being the most overexpressed protein at Day 24. ALB is a soluble, monomeric protein that comprises about one-half of blood serum protein. Albumin functions primarily as a carrier protein for steroids, fatty acids, and thyroid hormones and plays a role in stabilising extracellular fluid volume. PGAM1 catalyses the conversion of 3-PG to 2-PG, a crucial step in glycolysis [20]. Finally, IDH1 is an enzyme that converts isocitrate and NADP+ to α-ketoglutarate (αKG) and NADPH [21]. Comparing the identified proteins with those previously identified at Day 14 [7], ALB, HSPA8, HSPA5, PDIA3 and ACTB were also identified at Day 24. Interestingly, all these proteins were also altered in pre-eclampsia, a human placental dysfunction [22].

If we consider that at Day 24 all the live foetuses are going to be born, we might conclude that these altered proteins are not responsible for prenatal losses. Therefore, what could be the consequences of these alterations? On one hand, in our last work we observed that vitrified pups weighed more than controls at day of birth. This result has also been observed several times in human babies [23–25]. In rabbits, during the last week of gestation (from Day 24 till birth) the fastest increase in weight of the rabbit pups takes place. So, future works will determine if there is a direct relation between altered proteins, such as PGAM1 and ADK, related with rapid growth of tumour cells [26, 27] and an increased weight of vitrified pups. On the other hand, it could be suggested that these altered proteins might be involved in postnatal development and adulthood. It is important to highlight that effects of vitrification on foetal placenta proteome were observed more than 11 and 21 days after the embryo vitrification and manipulation. This could occur because a memory of the injury is stored, mainly by the presence of epigenetic marks. It has been reported previously that epigenetic information of the embryo could be modified due to vitrification process [28, 29]. As has been suggested, a number of organ structures and associated functions undergo programming during the embryonic stage, foetal life and neonatal period, which determines the set point of physiological and metabolic responses that are kept into adulthood [30, 31]. Further studies should focus on these epigenetic changes, and figure out if vitrification alterations could entail consequences not only in the last part of gestation, but also in adult life. A recent work observed how the females of a re-established rabbit population from vitrified embryo resulted in better reproductive traits in the F1 and F2 generation [32].

In conclusion, our results revealed proteomic disturbances in foetal placental tissues caused by vitrification procedure for the cryopreservation of embryos. This study shows for the first time that the proteome alterations remained during gestation. This effect, taken together with other effects reported in the literature, suggests that embryo cryopreservation is not neutral. Nevertheless, in order to evaluate long-term consequences of embryo vitrification, in future studies analysis of permanent tissues such as liver, kidney or brain must be performed to determine whether the effects observed during foetal development could lead to physiological and metabolic disorders in adulthood.
